# Role of adult hippocampal neurogenesis in the antidepressant actions of lactate

**DOI:** 10.1038/s41380-021-01122-0

**Published:** 2021-05-14

**Authors:** Anthony Carrard, Frédéric Cassé, Charline Carron, Sophie Burlet-Godinot, Nicolas Toni, Pierre J. Magistretti, Jean-Luc Martin

**Affiliations:** 1 Center for Psychiatric Neurosciences, Lausanne University Hospital, Lausanne, Switzerland; 2 King Abdullah University of Science and Technology (KAUST), Thuwal, Saudi Arabia; 3 University of Lausanne, Lausanne, Switzerland

**Keywords:** Neuroscience, Stem cells

## Abstract

In addition to its role as a neuronal energy substrate and signaling molecule involved in synaptic plasticity and memory consolidation, recent evidence shows that lactate produces antidepressant effects in animal models. However, the mechanisms underpinning lactate’s antidepressant actions remain largely unknown. In this study, we report that lactate reverses the effects of corticosterone on depressive-like behavior, as well as on the inhibition of both the survival and proliferation of new neurons in the adult hippocampus. Furthermore, the inhibition of adult hippocampal neurogenesis prevents the antidepressant-like effects of lactate. Pyruvate, the oxidized form of lactate, did not mimic the effects of lactate on adult hippocampal neurogenesis and depression-like behavior. Finally, our data suggest that conversion of lactate to pyruvate with the concomitant production of NADH is necessary for the neurogenic and antidepressant effects of lactate.

## Introduction

Post-mortem studies have shown that major depression is associated with a reduction in the number and density of astrocytes in various frontolimbic areas of depressed patients, suggesting that astrocyte dysfunction may contribute to the pathogenesis of depression [1–3]. Astrocytes are involved in essential CNS functions including energy metabolism, synaptic transmission, synaptic plasticity, and adult neurogenesis [4–7]. With regard to adult hippocampal neurogenesis, blockade of vesicular release from astrocytes impairs the synaptic maturation and survival of new neurons [8]. Compelling evidence supports the major contribution of astrocytes to neuroenergetics as they play a central role in energy production, storage, and delivery [4]. Upon glutamatergic neuronal activation, astrocytes respond by increasing their rate of glucose utilization and release of lactate that can be used as an energy substrate for neurons [9]. Lactate serves as an energy substrate via its oxidation to pyruvate, a reaction catalyzed by lactate dehydrogenase and coupled to NADH production. In addition, increasing evidence supports a role for lactate as a signaling molecule [4] involved, among other functions, in long-term memory formation [10, 11] and conditioned responses to cocaine [12, 13].

Previous studies from our laboratory have provided evidence indicating an antidepressant role of lactate. Indeed, fluoxetine and paroxetine increase lactate release by cortical astrocytes [14], and treatment of cortical neurons with lactate regulates the expression of plasticity-related genes involved in the pathophysiology and treatment of depression such as *BDNF*, *ARC*, and *VEGFA* [15, 16]. Furthermore, chronic peripheral administration of lactate produces antidepressant-like effects as evidenced by the reduction of behavioral despair and anhedonia-like behavior and by reversal of social avoidance [17, 18]. However, the mechanisms by which lactate produces its antidepressant effects are still unclear. Investigation of the molecular mechanisms underlying the chronic antidepressant effects of lactate has revealed that the expression of Hes5, a downstream effector of the Notch signaling pathway that plays a central role in adult hippocampal neurogenesis [19], is regulated by lactate [17]. In addition to Hes5, the expression of p11, a protein involved in the regulation of hippocampal neurogenesis by fluoxetine [20], is upregulated by lactate [17]. While chronic stress decreases adult hippocampal neurogenesis [21], chronic treatment with different classes of antidepressants increases adult hippocampal neurogenesis in rodents [22, 23]. In addition, adult hippocampal neurogenesis is required for some of the behavioral effects of SSRI and tricyclic antidepressants [24, 25]. Based on the above findings, we hypothesized that adult hippocampal neurogenesis may be involved in the mechanisms underlying the antidepressant actions of lactate.

## Materials and methods

### Drugs/reagents/antibodies

Sodium L-lactate, sodium L-pyruvate, temozolomide (TMZ), sodium β-hydroxybutyric acid, sesame oil, 5-Bromo-2′-deoxyuridine (BrdU), poly-L-ornithine, L-arginine, L-lysine, glucose, paraformaldehyde, DMSO, and saccharin were from Sigma-Aldrich (Switzerland). Corticosterone was from Tocris Bioscience (UK). β-Nicotinamide adenine dinucleotide (NADH) was from Roche (Switzerland). CellROX green reagent and 4′,6-diamidine-2′-phenylindole dihydrochloride (DAPI) were from Thermo Fisher Scientific (USA). Sodium acetoacetate was from BOC Sciences (USA). Penicillin and streptomycin were from Invitrogen (USA). SILAC advanced Dulbecco’s Modified Eagle’s Medium/F-12 flex media, N2 supplement, and mouse laminin were from Gibco (USA).

Anti-BrdU mouse monoclonal antibody (B44) was from BD Biosciences (USA). Goat anti-mouse Alexa Fluor 488 (A11029) and 594 (A11032) antibodies and goat anti-rabbit Alexa Fluor 488 (A11008) antibody were from Thermo Fisher Scientific (USA). Anti-NeuN rabbit monoclonal antibody (ab177487) was from Abcam (UK). Anti-SOX-2 rabbit polyclonal antibody (AB5603) and anti-Nestin mouse monoclonal antibody (MAB353) were from Sigma-Aldrich (Switzerland).

### Animals

Experiments were conducted in accordance with the Swiss Federal Guidelines for Animal Experimentation and were approved by the Cantonal Veterinary Office for Animal Experimentation (Vaud, Switzerland). Eight to ten-week-old male C57Bl/6 mice (Janvier Labs, France) were housed under a 12 h light-dark cycle at a temperature of 22 °C ± 2 °C with a relative humidity of 55% ± 10%. Mice had ad libitum access to water and a standard rodent chow diet.

### Animal treatments

An emulsion of corticosterone (4 mg/ml) was prepared by mixing corticosterone with 2% DMSO in sesame oil. Mice received a single subcutaneous injection of corticosterone (20 mg/kg) [17] or vehicle (2% DMSO in sesame oil) on each of every 21 consecutive days. In addition, corticosterone-treated mice were given intraperitoneal injections of vehicle (0.9% NaCl; 5 ml/kg i.p.), L-lactate (1 g/kg; 5 ml/kg i.p.) or L-pyruvate (1 g/kg; 5 ml/kg i.p.) daily for 21 days. For TMZ  administration, mice were treated on the first 3 days of a week for four 4 consecutive weeks with TMZ (25 mg/kg; 2.5 mg/ml in 0.9% NaCl i.p.) or saline (0.9% NaCl), as previously described [26]. 24 h after the last injection, mice were either subjected to the forced swim test, tail suspension test (TST), saccharin preference test or perfused with 4% PFA for BrdU and NeuN immunostaining. For the analysis of cell survival and proliferation, animals were injected with BrdU (100 mg/kg; 10 ml/kg i.p.) 24, 8, and 4 h before the sacrifice for cell proliferation analysis or the day before the beginning of the treatment with corticosterone (14 consecutive days) for cell survival analysis.

### Forced swim test

The forced swim test (FST) was performed as previously described [17]. Briefly, male C57Bl/6 mice were placed in a 5 L cylindrical container filled to a depth of 20 cm with water (23–25 °C). A 6 min swim test session was videotaped, and time spent immobile (defined as minimal movements necessary to stay afloat) was automatically scored using EthoVision XT 11.5 software (Noldus, The Netherlands). Time spent immobile during the swim session was scored 5 min after the initial minute.

### TST

The TST was performed as previously described [17]. Briefly, mice were suspended by the tail from a metal bar and videotaped for 5 min. Total immobility time was manually recorded by the experimenter blind to the testing conditions.

### Saccharin preference test

The saccharin preference test was performed according to a previously published procedure [17]. Twenty-four hours after the last drug administration, mice (housed 2 per cage) were presented with identical drinking bottles, one filled with water and the other one with 0.02% saccharin dissolved in water. The position of the two bottles was changed every 24 h. Bottles were weighed before and 72 h after the drinking session to determine the volume of the consumed fluid. Data were calculated as the ratio of the volume of saccharin consumed to the total volume of fluid consumed.

### Immunohistochemistry on brain tissue sections

At the end of the experiment, mice were deeply anesthetized with a lethal dose of pentobarbital, and brains were fixed by transcardial perfusion with 50 ml of 0.9% saline followed by 100 mL of 4% PFA dissolved in phosphate-buffered saline (PBS 0.1 M, pH 7.4). Brains were then removed, postfixed in 4% PFA for 24 h at 4 °C, cryoprotected 24 h in cryoprotectant solution (30% ethylene glycol and 30% sucrose in PBS), rapidly frozen and sectioned at a thickness of 40 µm with a microtome-cryostat (Leica CM3050S). To cover the whole dentate gyrus, one in every seven sections for a total of ten brain sections per animal was used for immunostaining. For visualization of BrdU incorporation, slices were incubated in formamide solution (50% formamide, 40% water, 10% SSC buffer 20X) for 2 h at 60 °C followed by DNA denaturation in 2 N HCl for 30 min at 37 °C and rinsed in 0.1 M sodium borate buffer (pH 8.5) for 15 min before being washed six times in PBS. Sections were incubated in blocking solution (PBS supplemented with 0.3% TritonX-100 and 10% horse serum) for 1 h at room temperature and then in blocking solution containing an anti-BrdU mouse monoclonal antibody (1:250) overnight at 4 °C. The following day, sections were washed in PBS and incubated for 1 h with a goat anti-mouse Alexa Fluor 594 antibody (1:300) in blocking solution. Nuclear staining was performed by incubating brain slices in PBS containing DAPI (2 μg/ml) for 1 h. For the analysis of adult neurogenesis in the dentate gyrus, brain sections were incubated with an anti-BrdU mouse monoclonal antibody (1:250) together with an anti-NeuN rabbit monoclonal antibody (1:1000) overnight at 4 °C as described above. The following day, brain sections were incubated with a goat anti-mouse Alexa Fluor 594 antibody (1:300) together with a goat anti-rabbit Alexa Fluor 488 antibody (1:300) for 1 h in blocking solution. BrdU^+^ cells were counted in the granule cell layer and subgranular zone (SGZ) with an Axioscope II (Carl Zeiss) fluorescence microscope. Representative images for illustrations and for BrdU colocalization with the neuronal marker NeuN were acquired using a confocal microscope (Zeiss LSM 780 Quasar Carl Zeiss, Oberkochen, Germany). To quantify the density of BrdU^+^ NeuN^+^ cells, confocal images were taken every seven sections throughout the entire granular cell layer of the dentate gyrus. Ten sections per animal were analyzed to cover the whole dentate gyrus. The surface area of the granule cell layer was outlined and measured using ImageJ software (NIH). To obtain the volume of the granule cell layer, the surface area was multiplied by the thickness of the slice (40 μM). The density of BrdU^+^ NeuN^+^ cells was obtained by dividing the number of BrdU^+^ NeuN^+^ cells by the corresponding volume of the granule cell layer.

### Adult hippocampal neural stem/progenitor cell culture

Adult rat hippocampal neural stem/progenitor cells were a kind gift from the laboratory of Prof. F. Gage, Salk Institute, San Diego, USA [27]. They were cultured in SILAC advanced Dulbecco’s Modified Eagle’s Medium/F-12 flex media supplemented with N2, 5 mM glucose, 0.7 mM L-arginine, 0.5 mM L-lysine, penicillin (100 IU/ml), and streptomycin (100 mg/ml). Twenty-four-well cell culture plates (TPP) were coated with poly-L-ornithine (10 μg/ml) and mouse laminin (5 μg/ml). Adult hippocampal neural stem/progenitor cells were plated at a density of 50,000 cells per well and treated for 2 days starting 6 h after plating. Characterization of neural stem/progenitor cell cultures by immunostaining with the specific neural stem cell markers Nestin and SOX-2 revealed that 97.3 ± 0.01% and 99.6 ± 0.04% of total neural stem/progenitor cells were positive for Nestin and SOX-2, respectively (Fig. S1).

### BrdU immunostaining in adult hippocampal neural stem/progenitor cell culture

For BrdU immunostaining, stem/progenitor cells were incubated with BrdU (5 μM) for 30 min after which the medium was replaced with fresh medium and cells were cultured for one hour. Cells were then fixed with 4% PFA and subjected to immunostaining. To make BrdU accessible to the antibody, stem/progenitor cell DNA was denatured in 2 N HCl for 15 min at 37 °C. After rinsing with 0.1 M borate buffer pH 8.5 for 15 min and three times with PBS for 5 min, stem/progenitor cells were incubated in blocking solution for 1 h and then in blocking solution containing an anti-BrdU mouse monoclonal antibody (1:250) overnight at 4 °C. The following day, stem/progenitor cells were washed with PBS and incubated with a goat anti-mouse Alexa Fluor 488 antibody (1:300) in a blocking solution for 1 h. Images were taken with an inverted fluorescence microscope (Nikon, Eclipse Ti2-E).

### CellROX assay

CellROX assay was performed according to the manufacturer’s instructions (Thermo Fisher Scientific, USA). This assay uses a cell-permeant fluorogenic ROS sensor (CellROX^TM^ green reagent) that is nonfluorescent in its reduced state and that exhibits a strong fluorescence upon oxidation within the cell. At the end of the treatment, stem/progenitor cells were incubated with CellROX green reagent (5 μM) and NucBlue (one drop per well) for 30 min at 37 °C before being live imaged with an inverted fluorescence microscope (Nikon, Eclipse Ti2-E) in an environmental control chamber (37 °C, 5% CO_2_). CellROX fluorescence intensity per cell was analyzed using ImageJ software (NIH).

### Statistical analysis

Data were presented as mean ± SEM. The normal distribution and homogeneity of variances were assessed using Shapiro-Wilk and Bartlett’s tests, respectively. The sample size was determined to obtain a power of at least 0.8 using Gpower Analysis Software (v3.1.9.2 Düsseldorf University, Germany). For all experiments, Student’s *t*-test or one-way ANOVA followed by Tukey post-hoc test was performed. Although Tukey post-hoc test is less conservative, it allows pairwise comparisons between groups with different sample sizes and represents a good compromise for intra-group analysis. Statistical analyses were performed with StatView 5.0 (SAS Institute, NC, USA), using an alpha level of 0.05.

## Results

Behavioral studies have shown that chronic administration of corticosterone induces depression-like states in rodents [28, 29]. We recently found that chronic peripheral administration of lactate suppresses the effects of corticosterone on behavioral despair and anhedonia [17].

**Fig. 1:**
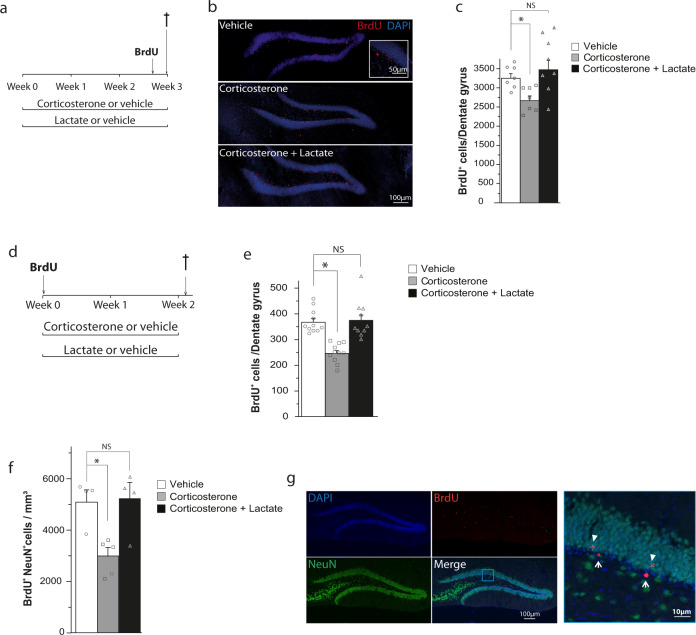
Peripheral administration of lactate reverses the reduction of adult hippocampal neural progenitor cells (NPCs) proliferation and survival in the corticosterone model of depression. **a** *Timeline showing the experimental design for measuring NPCs proliferation*. Mice received a single subcutaneous injection of corticosterone (20 mg/kg) or vehicle (2% DMSO in sesame oil) on each of 21 consecutive days. Together with corticosterone treatment, mice received intraperitoneal injections of vehicle (0.9% NaCl) or lactate (1 g/kg) daily for 21 days. On the last day of treatment, mice also received BrdU injections. **b** *Confocal maximal projection micrographs of hippocampal sections immunostained for BrdU*. Inset: higher magnification confocal micrograph of a BrdU-immunolabeled group of cells. **c** *Analysis of NPCs proliferation*. Histogram of the number of BrdU^+^ cells in the granule cell layer of the dentate gyrus. **d** *Timeline showing the experimental design for measuring NPCs survival*. BrdU administration was performed the day before the start of the treatment. Then, mice received a single subcutaneous injection of corticosterone (20 mg/kg) or vehicle (2% DMSO in sesame oil) on each of 14 consecutive days. Together with corticosterone treatment, mice received intraperitoneal injections of vehicle (0.9% NaCl) or lactate (1 g/kg) daily for 14 days. **e** *Analysis of NPCs survival*. Histogram of the number of BrdU^+^ cells in the granule cell layer of the dentate gyrus. **f** *Analysis of adult neurogenesis in the mouse dentate gyrus*. Histogram showing the number of BrdU^+^ NeuN^+^ cells per mm^3^. **g** *Confocal images of cells double-labeled for BrdU (red) and NeuN (green) and counterstained with DAPI (blue) in the dentate gyrus*. The right panel corresponds to a higher magnification view of the boxed region shown in the merged image. Solid arrows point to BrdU^+^ NeuN^+^ cells and thin arrows to BrdU^+^ cells. Data are the mean ± SEM. One-way ANOVA followed by Tukey post-hoc test ((**c**): *n* = 7/condition; (**e**): *n* > 10/condition; (**f**): *n* > 4/condition). *p < 0.05. NS not significant.

As a first step toward assessing the role of adult hippocampal neurogenesis in the antidepressant effects of lactate, we investigated whether chronic peripheral administration of lactate reverses the effects of corticosterone on cell proliferation in the SGZ of the dentate gyrus. To this end, animals were treated with either vehicle, corticosterone, or both corticosterone and lactate for 3 weeks and injected with BrdU within the first 24 h after the last drug administration (Fig. 1a). Consistent with previous findings [30, 31], chronic corticosterone administration increased immobility in the FST (Fig. S2) and decreased cell proliferation in the SGZ (Fig. 1b, c). In contrast, chronic treatment with lactate reversed the effects of corticosterone both on the immobility in the FST (Fig. S2b) and on the decreased SGZ cell proliferation (Fig. 1b, c).

Adult neurogenesis is a continuous process and a temporary treatment may also affect the survival of neurons that were generated before treatment initiation. To test the effect of lactate on the survival of cells that divided before treatment initiation, animals were injected with BrdU before treatment. Animals were then injected with either vehicle, corticosterone, or both corticosterone and lactate (Fig. 1d). While chronic administration of corticosterone decreased the survival of cells formed just before treatment, lactate restored the survival of these cells to vehicle values (Fig. 1e). It is worth noting that chronic lactate administration did not affect cell proliferation nor survival in animals not treated with corticosterone (Fig. S3a–d). Thus, lactate reversed the effects of corticosterone on behavioral despair and on cell proliferation as well as cell survival in the SGZ of the dentate gyrus.

Next, we examined whether corticosterone and lactate administration regulated adult hippocampal neurogenesis. Consistent with the data on cell proliferation and survival (Fig. 1c, e), corticosterone administration reduced the density of BrdU+ cells that express the mature neuronal marker NeuN in the dentate gyrus (Fig. 1f), indicating that chronic corticosterone treatment inhibits adult hippocampal neurogenesis. In contrast, lactate administration reversed the inhibitory effect of corticosterone on the density of BrdU+ NeuN+ cells in the dentate gyrus (Fig. 1f), which is in line with its counteracting effects on cell proliferation and survival (Fig. 1c, e).

**Fig. 2:**
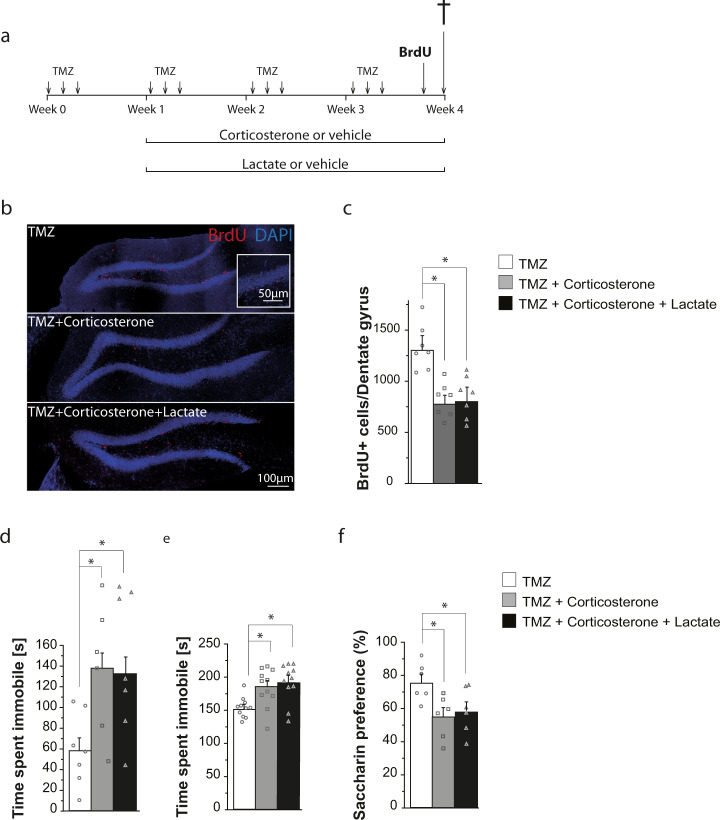
Inhibition of adult hippocampal neurogenesis by temozolomide suppresses the antidepressant and neurogenic effects of lactate in the corticosterone model of depression. **a** *Timeline showing the experimental design*. Mice received an intraperitoneal injection of temozolomide (TMZ) (25 mg/kg) on the first 3 days of a week for 4 consecutive weeks. On the second week of TMZ treatment, mice received a daily subcutaneous injection of vehicle or corticosterone (20 mg/kg) and an intraperitoneal injection of vehicle (NaCl 0.9%) or lactate (1 g/kg) for 21 consecutive days. On the last day of treatment, mice received BrdU injections. **b** *Confocal maximal projection micrographs of hippocampal sections immunostained for BrdU*. Inset: Higher magnification confocal micrograph of a BrdU-immunolabeled group of cells. **c** *Analysis of neural progenitor cells (NPCs) proliferation*. Histogram of the number of BrdU^+^ cells in the granule cell layer of the dentate gyrus. **d** *Assessment of depressive-like behavior in FST*. Histogram of the time spent immobile during FST. **e** *Assessment of depressive-like behavior in TST*. Histogram of the time spent immobile during TST. **f** *Assessment of anhedonia-like behavior in the saccharin preference test*. Data are the mean ± SEM. One-way ANOVA followed by Tukey post-hoc test ((**c**): *n* = 7/condition; (**d**): *n* = 8/condition; (**e**): *n* = 11/condition, (**f**): *n* = 6/condition). *p < 0.05.

To determine whether adult hippocampal neurogenesis is required for the antidepressant actions of lactate, we used TMZ, an antimitotic drug that impairs hippocampal neurogenesis [26]. While reducing adult hippocampal neurogenesis by TMZ did not prevent the decreased cell proliferation induced by corticosterone, it suppressed the counteracting effect of lactate on cell proliferation in corticosterone-treated animals (Fig. 2b, c). In addition, depletion of adult hippocampal neurogenesis by TMZ abolished the antidepressant effects of lactate in the corticosterone model of depression (Fig. 2d–f). Thus, chronic administration of lactate in animals treated with TMZ did not prevent the increased immobility induced by corticosterone in the FST (Fig. 2d) and TST (Fig. 2e) and did not reverse the corticosterone-induced decrease in saccharin preference (Fig, 2f). Of note, mice injected with TMZ did not show alterations in body weight, locomotor activity, and neuromuscular strength (Fig. S4a–d). Together, these data suggest that adult hippocampal neurogenesis is required for the antidepressant effects of lactate in the corticosterone model of depression.

**Fig. 3:**
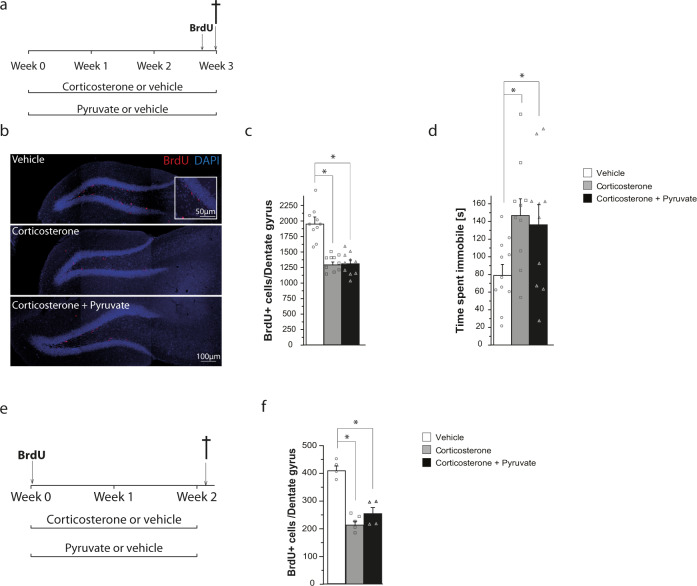
Lack of antidepressant and neurogenic effects of pyruvate in the corticosterone model of depression. **a** *Timeline showing the experimental design for measuring neural progenitor cells (NPCs) proliferation*. Mice received a single subcutaneous injection of corticosterone (20 mg/kg) or vehicle (2% DMSO in sesame oil) on each of 21 consecutive days. Together with corticosterone treatment, mice received intraperitoneal injections of vehicle (0.9% NaCl) or pyruvate (1 g/kg) daily for 21 days. On the last day of treatment, mice also received BrdU injections. **b** *Confocal maximal projection micrographs of hippocampal sections immunostained for BrdU*. Inset: Higher magnification confocal micrograph of a BrdU-immunolabeled group of cells. **c** *Analysis of NPCs proliferation*. Histogram of the number of BrdU^+^ cells in the granule cell layer of the dentate gyrus. **d** *Assessment of depressive-like behavior in FST*. Histogram of the time spent immobile during FST. **e** *Timeline showing the experimental design for measuring NPCs survival*. BrdU administration was performed the day before the start of the treatment. Then, mice received a single subcutaneous injection of corticosterone (20 mg/kg) or vehicle (2% DMSO in sesame oil) on each of 14 consecutive days. Together with corticosterone treatment, mice received intraperitoneal injections of vehicle (0.9% NaCl) or pyruvate (1 g/kg) daily for 14 days. **f** *Analysis of NPCs survival*. Histogram of the number of BrdU^+^ cells in the granule cell layer of the dentate gyrus. Data are the mean ± SEM. One-way ANOVA followed by Tukey post-hoc test ((**c**), (**d**): *n* > 10/condition; (**f**): *n* = 4/condition). *p < 0.05.

Lactate is used as an energy substrate via its oxidation to pyruvate. Conversion of lactate to pyruvate is catalyzed by lactate dehydrogenase and is coupled to NADH production. Both lactate and pyruvate are transported across the blood-brain barrier by monocarboxylate transporter 1 [32]. To investigate whether pyruvate may have antidepressant effects, animals were treated with either vehicle, corticosterone, or both corticosterone and pyruvate for 3 weeks and subjected to FST (Fig. 3a). In marked contrast to lactate, chronic administration of pyruvate did not produce antidepressant-like effects (Fig. 3d). Thus, chronic pyruvate treatment did not reverse the increased immobility induced by corticosterone in the FST (Fig. 3d). In addition to its lack of antidepressant effect, pyruvate did not reverse the decreased proliferation (Fig. 3b, c) and survival (Fig. 3f) of newly-formed cells in the SGZ of the dentate gyrus of animals treated with corticosterone, indicating that pyruvate does not affect adult hippocampal neurogenesis in the chronic corticosterone paradigm.

Our observations that lactate and pyruvate exhibit contrasting effects suggest that NADH generated through the oxidation of lactate to pyruvate by lactate dehydrogenase may participate in the neurogenic and antidepressant actions of lactate.

**Fig. 4:**
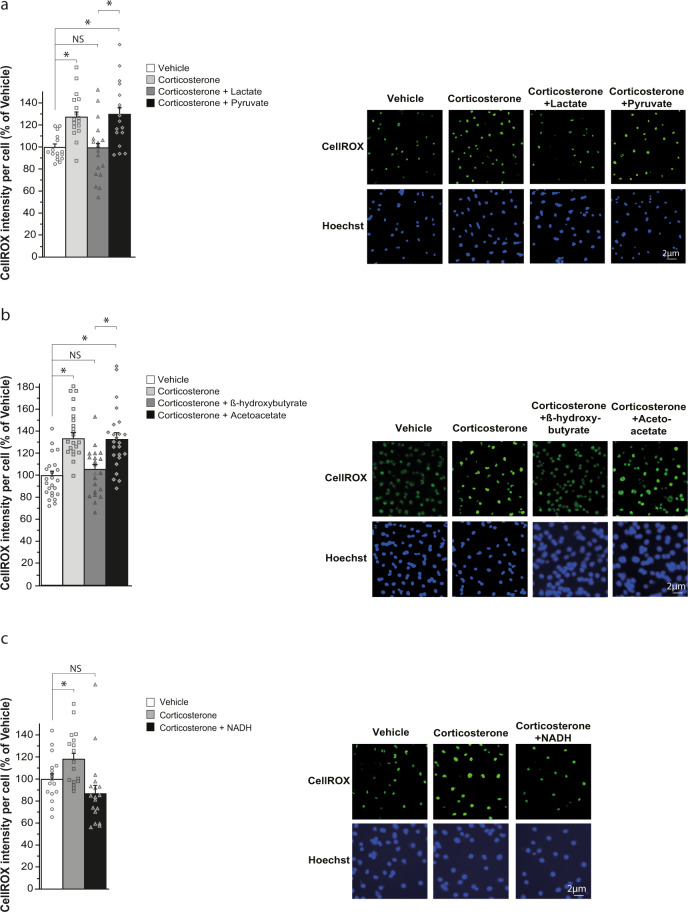
Lactate, β-hydroxybutyrate, and NADH suppress ROS production induced by corticosterone in adult hippocampal stem/progenitor cells in vitro. *Analysis of ROS production*. Cultures of adult hippocampal stem/progenitor cells were treated for 48 h with corticosterone (5 μM) together with either lactate (20 mM) (**a**), pyruvate (20 mM) (**a**), β-hydroxybutyrate (20 mM) (**b**), acetoacetate (20 mM) (**b**) or NADH (100 μM) (**c**). Left panels: histograms of CellROX intensity per cell shown as the percentage of the vehicle. Right panels: illustrations of CellROX fluorescence (green) in stem/progenitor cells counterstained with NucBlue (Hoechst)(blue). Data are the mean ± SEM. One-way ANOVA followed by Tukey post-hoc test (*n* > 17/condition). *p < 0.05. NS not significant.

**Fig. 5:**
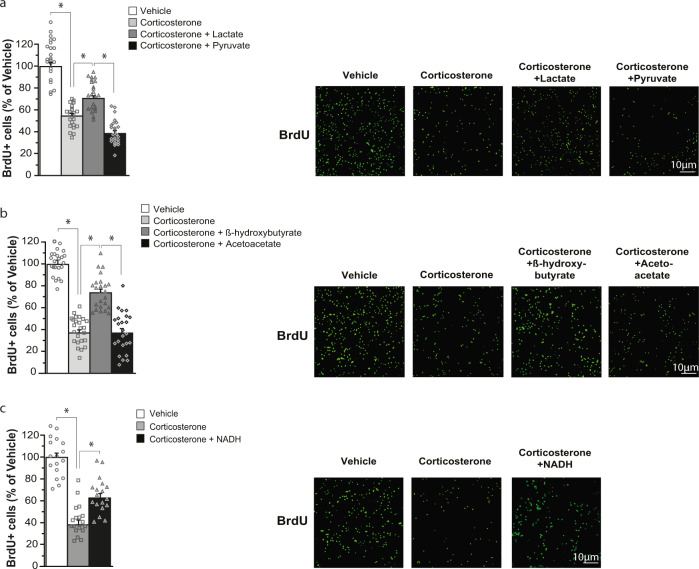
Lactate, β-hydroxybutyrate, and NADH partially counteract the decreased proliferation of adult hippocampal stem/progenitor cells induced by corticosterone in vitro. *Analysis of stem/progenitor cell proliferation*. Cultures of adult hippocampal stem/progenitor cells were treated for 48 h with corticosterone (5 μM) together with either lactate (20 mM) (**a**), pyruvate (20 mM) (**a**), β-hydroxybutyrate (20 mM) (**b**), acetoacetate (20 mM) (**b**) or NADH (100 μM) (**c**). Left panels: histograms of the number of BrdU^+^ cells presented as a percentage of the vehicle. Right panels: fluorescent images of stem/progenitor cells immunostained for BrdU. Data are the mean ± SEM. One-way ANOVA followed by Tukey post-hoc test (*n* > 16/condition). *p < 0.05.

Previous in vitro and in vivo studies have shown that corticosterone increases neuronal oxidative stress [33–35]. This led us to examine whether lactate inhibited reactive oxygen species (ROS) production induced by corticosterone in adult hippocampal stem/progenitor cell culture. To this aim, cells were treated with corticosterone or both corticosterone and lactate and ROS production was assessed using CellROX green reagent. Treatment of hippocampal stem/progenitor cells with corticosterone increased ROS production and, co-treatment with lactate suppressed the increased generation of ROS by corticosterone (Fig. 4a). Furthermore, lactate partially reversed the effect of corticosterone on the inhibition of stem/progenitor cells proliferation (Fig. 5a). In contrast, pyruvate did not attenuate the effects of corticosterone on ROS production and stem/progenitor cell proliferation (Figs. 4a and  5a). Interestingly, β-hydroxybutyrate, a ketone body transported by the same monocarboxylate transporters as lactate and pyruvate [32], has been shown to produce antidepressant-like effects [36]. Based on these findings, we examined whether β-hydroxybutyrate and its oxidized form, acetoacetate, could interfere with the effects of corticosterone on ROS production and stem/progenitor cell proliferation. We observed that β-hydroxybutyrate, like lactate, suppressed the effect of corticosterone on ROS production (Fig. 4b) and partially counteracted the effect of corticosterone on stem/progenitor cells proliferation (Fig. 5b), whereas acetoacetate had no effect (Figs. 4b and 5b).

As the conversion of lactate into pyruvate and of β-hydroxybutyrate into acetoacetate by lactate dehydrogenase and β-hydroxybutyrate dehydrogenase, respectively, is coupled to NADH production, we assessed whether NADH could counteract the effects of corticosterone on ROS production and stem/progenitor cell proliferation. Treatment of hippocampal stem/progenitor cells with NADH prevented ROS production elicited by corticosterone (Fig. 4c) and partially reversed the inhibition of stem/progenitor cell proliferation induced by corticosterone (Fig. 5c). Overall, our data indicate that oxidation of lactate into pyruvate with the concomitant production of NADH is necessary for the neurogenic effects of lactate and suggests that they are mediated, in part, by the suppression of ROS generated by corticosterone.

## Discussion

In this study, we examined the involvement of adult hippocampal neurogenesis in the antidepressant-like effect of lactate on corticosterone-induced depressive-like behavior in mice. We report that lactate, but not pyruvate, reverses the effects of corticosterone on the survival and proliferation of adult-born hippocampal neurons. In addition, we found that inhibiting adult hippocampal neurogenesis prevents the antidepressant-like effects of lactate. The effects of lactate on stem/progenitor cells may be mediated by counteracting ROS production induced by corticosterone.

Recent data from our laboratory have shown that peripheral administration of lactate produces antidepressant-like effects in different animal models of depression that respond to acute and chronic antidepressant treatment [17]. Interestingly, evidence supporting the antidepressant actions of lactate was recently extended to other animal models of depression. Indeed, chronic intraperitoneal injections of lactate reverse social avoidance behavior in the chronic social defeat stress paradigm [18]. In addition, oral lactate administration produces antidepressant-like effects in an animal model of menopausal depression [37].

Investigation of the molecular mechanisms underlying the chronic antidepressant effects of lactate has revealed that lactate regulates the expression of a specific group of genes among which Hes5 is involved in adult hippocampal neurogenesis [17]. In this context, rodent studies have provided evidence supporting the role of adult hippocampal neurogenesis in the action of certain antidepressants [21]. For instance, ablation of hippocampal neurogenesis suppresses the efficacy of fluoxetine in some behavioral paradigms, suggesting the existence of neurogenesis-dependent mechanisms of antidepressant actions [25]. The role of adult hippocampal neurogenesis in the antidepressant effects of lactate was examined in the corticosterone model of depression that reproduces behavioral and neurobiological changes associated with human depression such as anhedonia and dysregulated HPA function [29]. Consistent with previous findings [38, 39], we found that chronic corticosterone administration decreased adult hippocampal neurogenesis in rodents (Fig. 1f). In contrast, chronic lactate treatment reversed the inhibitory effect of corticosterone on the proliferation (Fig. 1c) and survival (Fig. 1e) of adult-born hippocampal neurons.

Interestingly, recent data from our laboratory indicate that lactate upregulates pro-survival genes and downregulates pro-death genes in cultured cortical neurons [16]. This suggests that the survival-promoting effects of lactate may be involved in the reversal effect of lactate on the survival of adult-generated hippocampal neurons in animals treated with corticosterone (Fig. 1e). In animals not treated with corticosterone, chronic administration of lactate did not affect cell proliferation and survival (Fig. S3a–d). These findings are in line with the observation that adult hippocampal neurogenesis is most consistently increased by antidepressants in stressed animals [25, 40]. Although lactate has been recently shown to promote adult hippocampal neurogenesis after prolonged exposure of 7 weeks [41], it is worth noting that this effect was not due to the stimulation of progenitor cell proliferation but rather to increased survival of newly born mature neurons.

Similar to lactate, different types of antidepressants counteract the effects of chronic corticosterone administration on the reduction of progenitor cell proliferation. For instance, previous studies have shown that fluoxetine reverses the decreased proliferation of hippocampal progenitor cells induced by chronic corticosterone treatment [25, 42–44]. In addition, agmatine, creatine, and agomelatine, which produce antidepressant-like effects in the corticosterone model of depression [45–47], reverse the effects of corticosterone on the proliferation of adult progenitors [42–44]. Similar to lactate (Fig. S3a–d), chronic administration of agomelatine, creatine, or fluoxetine does not affect hippocampal progenitor proliferation in non-corticosterone treated animals [25, 43, 44]. Together, these observations indicate that reversal of corticosterone-induced impairment of hippocampal progenitor proliferation contributes to the action of various types of antidepressants.

Inhibition of adult hippocampal neurogenesis suppresses the antidepressant effects of lactate both on behavioral despair (Fig. 2d, e) and anhedonia (Fig. 2f). Interestingly, the SSRI antidepressant fluoxetine produces neurogenesis-dependent and -independent behavioral effects in animals chronically treated with corticosterone [25]. In contrast to lactate (Fig. 2d), ablation of adult hippocampal neurogenesis in mice chronically treated with corticosterone did not affect the antidepressant effect of fluoxetine in the FST [25]. However, unlike our study (Fig. 2d), chronic corticosterone administration did not induce depressive-like behavior in the FST [25].

The effects of lactate on the proliferation and survival of adult-born hippocampal neurons markedly contrast with those of its oxidized form, i.e., pyruvate. Indeed, chronic administration of pyruvate does not produce antidepressant-like effects (Fig. 3d), nor reverses the effects of corticosterone on proliferation and survival of new neurons in the adult hippocampus (Fig. 3b, c, f).

Our observations that lactate and pyruvate have contrasting antidepressant and neurogenic actions (Figs. 1 and 3) suggest that the effects of lactate do not depend on its utilization as an energy substrate. Indeed, to serve as an energy substrate, lactate must first be converted to pyruvate, implying that lactate and pyruvate would have similar antidepressant and neurogenic effects. Conversion of lactate to pyruvate is catalyzed by lactate dehydrogenase with the concomitant reduction of NAD^+^ to NADH. Our data on adult hippocampal stem/progenitor cells in vitro suggest that the production of NADH by lactate oxidation participates in the neurogenic actions of lactate. Consistent with this idea, lactate, and NADH but not pyruvate suppress the effect of corticosterone on ROS production (Fig. 4a, c) and partially reverses the decreased proliferation of adult hippocampal stem/progenitor cells induced by corticosterone (Fig. 5a, c). As NADH maintains or restores cellular redox homeostasis [48], this suggests that treatment of hippocampal stem/progenitor cells with lactate or NADH reverses the effects of corticosterone on oxidative stress and thereby maintains cellular redox homeostasis. Thus, NADH produced by oxidation of lactate to pyruvate contributes to the effect of lactate on restoring adult hippocampal neurogenesis after corticosterone treatment possibly by re-establishing cellular redox homeostasis.

Interestingly, the observation that lactate suppresses the effects of corticosterone on ROS production (Fig. 4a) is consistent with previous findings demonstrating that NADH produced by conversion of lactate to pyruvate neutralizes ROS generation induced by glutamate in the hippocampus [49].

Regarding the mechanisms underlying the effects of lactate, we have previously found that lactate but not pyruvate potentiates NMDAR activity in cortical neurons through increases in intracellular NADH levels [15, 50]. In addition, we have shown that lactate but not pyruvate upregulates the expression of plasticity-related genes by a mechanism that depends on NADH and NMDAR activity [15, 16]. These data indicate that conversion of lactate to pyruvate with the concomitant production of NADH potentiates NMDAR activity. Upregulation of NMDAR activity by NADH may occur via NADH dehydrogenase subunit 2 that enables Src kinase regulation of NMDA receptors [51]. Interestingly, NMDA receptor activation regulates adult hippocampal neurogenesis. For instance, stimulation of NMDA receptors promotes adult hippocampal neurogenesis [52], supporting the idea that upregulation of NMDAR activity by NADH formed by oxidation of lactate to pyruvate mediates the reversal effect of lactate on the decreased proliferation and survival of adult-born hippocampal neurons induced by corticosterone treatment (Fig. 1). One should also note, however, that other studies have shown that the pharmacological blockade of NMDA receptors in vivo increases the proliferation of precursors in the rodent and tree shrew dentate gyrus [53, 54].

Although the clinical relevance of human adult neurogenesis has been debated [55–57], recent postmortem human studies have shown a smaller dentate gyrus volume, with fewer granule neurons and a lower number of neural progenitor cells in the anterior and mid dentate gyrus of MDD patients who committed suicide, suggesting an impaired proliferation and maturation of neural progenitor cells in depressed patients [58]. In contrast, MDD patients treated with SSRIs or TCAs have more hippocampal neural progenitor cells than untreated MDD patients and control subjects, supporting a possible role of adult hippocampal neurogenesis in the mechanisms underlying the antidepressant actions in MDD patients [59, 60]. Together, these data support a role for human adult hippocampal neurogenesis in depression and antidepressant response.

Several lines of evidence support a role for lactate as an energy substrate for neurons, a signaling molecule in synaptic plasticity, and more recently as an antidepressant in animal models [4, 10, 17]. The current study aimed at identifying the mechanisms of antidepressant actions of lactate shows that lactate restores adult hippocampal neurogenesis in a model of depressive-like behavior and that the antidepressant effect of lactate requires adult neurogenesis. Furthermore, our data suggest that conversion of lactate to pyruvate with the concomitant production of NADH is necessary for the neurogenic and antidepressant effects of lactate.

## Supplementary information

Supplementary figure 1 (Fig. S1)

Supplementary figure 2 (Fig. S2)

Supplementary figure 3 (Fig. S3)

Supplementary figure 4 (Fig. S4)

Supplementary figure legends
