# Supplementary material for: Role of adult hippocampal neurogenesis in the antidepressant actions of lactate
Source: Mol Psychiatry. 2021 May 14;26(11):6723–35. doi: 10.1038/s41380-021-01122-0 (PMC8760055; doi:10.1038/s41380-021-01122-0)
Supplement: Supplementary file 5 — Supplementary figure legends [file 41380_2021_1122_MOESM5_ESM.pdf]

Supplementary figure 1 (Fig S1).
Immunocytochemical characterization of adult rat hippocampal neural stem/progenitor cell cultures.
(a) Rat hippocampal stem/progenitor cells (6×10^4) cultured in 24-well plates were fixed with 4% PFA, immunostained with SOX-2 and Nestin antibodies, and nuclear stained with DAPI. (b) Percentage of SOX-2- and Nestin-positive cells compared to the total number of cells quantified by DAPI nuclear staining (n =8).

Supplementary figure 2 (Fig S2).
Chronic peripheral administration of lactate induces antidepressant-like effects in the corticosterone model of depression.
(a) *Timeline showing the experimental design*. Mice received a single subcutaneous injection of corticosterone (20 mg/kg) or vehicle (2% DMSO in sesame oil) on each of 21 consecutive days. Together with corticosterone treatment, mice received intraperitoneal injections of vehicle (0.9% NaCl) or lactate (1 g/kg) daily for 21 days. (b) *Assessment of depressive-like behavior in FST*. Histogram of the time spent immobile during FST. Data are the mean ± SEM. One-way ANOVA followed by Tukey post-hoc test (n>6/condition). *$p < 0.05$. NS, not significant.

Supplementary figure 3 (Fig S3).
Chronic peripheral administration of lactate does not affect neural progenitor cells (NPCs) proliferation and survival.
(a) *Timeline showing the experimental design for measuring NPCs proliferation.* Mice received a single subcutaneous injection of vehicle (2% DMSO in sesame oil) on each of 21 consecutive days. In addition, mice received intraperitoneal injections of vehicle (0.9% NaCl) or lactate (1 g/kg) daily for 21 days. On the last day of treatment, mice received BrdU injections. (b) *Analysis of NPCs proliferation*. Histogram of the number of $BrdU^+$ cells in the granule cell layer of the dentate gyrus. (c) *Timeline showing the experimental design for measuring NPCs survival.* BrdU administration was performed the day before the start of the treatment. Mice received a single subcutaneous injection of vehicle (2% DMSO in sesame oil) on each of 14 consecutive days. In addition, mice received intraperitoneal injections of vehicle (0.9% NaCl) or lactate (1 g/kg) daily for 14 days. (d) *Analysis of NPCs survival*. Histogram of the number of $BrdU^+$ cells in the granule cell layer of the dentate gyrus. Data are the mean ± SEM. Statistical analysis was performed using Student's t-test (n=7/condition). NS, not significant.

Supplementary figure 4 (Fig S4).
Temozolomide treatment does not affect body weight, neuromuscular strength and locomotor activity.
(a) *Timeline showing the experimental design for measuring the effects of temozolomide (TMZ) on body weight, neuromuscular strength and locomotor activity*. Mice were treated on the first three days of a week for 4 consecutive weeks with TMZ (25 mg/kg) or vehicle (0.9% NaCl). (b) Body weight was measured once a week. Data are the mean ± SEM. Repeated measures ANOVA (n=12/condition). Body weight was not significantly different between TMZ- and vehicle-treated mice. (c) Four days after the last injection, grip strength was measured, as previously described [17]. Data are the mean ± SEM. Student's t-test (n=12/condition). NS, not

significant. (d) Four days after the last injection, locomotor activity was measured, as previously described [17]. Data are the mean ± SEM. Student's t-test (n=12/condition). NS, not significant.
